# Genomic Comparison Among Global Isolates of *L. interrogans* Serovars Copenhageni and Icterohaemorrhagiae Identified Natural Genetic Variation Caused by an Indel

**DOI:** 10.3389/fcimb.2018.00193

**Published:** 2018-06-19

**Authors:** Luciane A. Santos, Haritha Adhikarla, Xiting Yan, Zheng Wang, Derrick E. Fouts, Joseph M. Vinetz, Luiz C. J. Alcantara, Rudy A. Hartskeerl, Marga G. A. Goris, Mathieu Picardeau, Mitermayer G. Reis, Jeffrey P. Townsend, Hongyu Zhao, Albert I. Ko, Elsio A. Wunder

**Affiliations:** ^1^Department of Epidemiology of Microbial Diseases, Yale School of Public Health, New Haven, CT, United States; ^2^Gonçalo Moniz Institute, Oswaldo Cruz Foundation, Salvador, Brazil; ^3^Department of Biostatistics, Yale School of Public Health, New Haven, CT, United States; ^4^J. Craig Venter Institute, Rockville, MD, United States; ^5^Division of Infectious Diseases, Department of Medicine, University of California San Diego School of Medicine, La Jolla, CA, United States; ^6^Royal Tropical Institute, KIT Biomedical Research, Amsterdam, Netherlands; ^7^Biology of Spirochetes Unit, Institut Pasteur, Paris, France

**Keywords:** *Leptospira*, leptospirosis, Copenhageni, Icterohaemorrhagiae, whole-genome sequencing, SNPs, Indels, phylogeny

## Abstract

Leptospirosis is a worldwide zoonosis, responsible for more than 1 million cases and 60,000 deaths every year. Among the 13 pathogenic species of the genus *Leptospira*, serovars belonging to *L. interrogans* serogroup Icterohaemorrhagiae are considered to be the most virulent strains, and responsible for majority of the reported severe cases. Serovars Copenhageni and Icterohaemorrhagiae are major representatives of this serogroup and despite their public health relevance, little is known regarding the genetic differences between these two serovars. In this study, we analyzed the genome sequences of 67 isolates belonging to *L. interrogans* serovars Copenhageni and Icterohaemorrhagiae to investigate the influence of spatial and temporal variations on DNA sequence diversity. Out of the 1072 SNPs identified, 276 were in non-coding regions and 796 in coding regions. Indel analyses identified 258 indels, out of which 191 were found in coding regions and 67 in non-coding regions. Our phylogenetic analyses based on SNP dataset revealed that both serovars are closely related but showed distinct spatial clustering. However, likelihood ratio test of the indel data statistically confirmed the presence of a frameshift mutation within a homopolymeric tract of *lic12008* gene (related to LPS biosynthesis) in all the *L. interrogans* serovar Icterohaemorrhagiae strains but not in the Copenhageni strains. Therefore, this internal indel identified can genetically distinguish *L. interrogans* serovar Copenhageni from serovar Icterohaemorrhagiae with high discriminatory power. To our knowledge, this is the first study to identify global sequence variations (SNPs and Indels) in *L. interrogans* serovars Copenhageni and Icterohaemorrhagiae.

## Background

Leptospirosis is a zoonosis with worldwide distribution and considered endemic in developing countries and tropical regions. This life-threatening disease is caused by pathogenic spirochetes from the genus *Leptospira* (Bharti et al., 2003; Mcbride et al., 2005; Ko et al., 2009). Globally, leptospirosis is conservatively estimated to cause 1.03 million cases and 58,900 deaths each year (Costa et al., 2015). Transmission of leptospirosis requires continuous enzootic circulation of the pathogen among animal reservoirs. This zoonosis is maintained in nature through chronic renal infection of carrier animals, with rodents and other small mammals being the most important reservoirs. Humans and other accidental hosts get infected by direct contact with infected animals or with contaminated water or soil (Bharti et al., 2003; Ko et al., 2009).

Pathogenic *Leptospira* includes 13 species and have been classified into over 250 distinct serotypes (Reis et al., 2008; Lehmann et al., 2014; Picardeau, 2017). In humans, severe leptospirosis is frequently associated with *L. interrogans* serogroup Icterohaemorrhagiae (Bharti et al., 2003). Currently majority of the human cases caused by Icterohaemorrhagiae serogroup are attributed to serovar Copenhageni strains. Although *Rattus norvegicus* is recognized as the main reservoir for serovar Copenhageni, rodents of the *Rattus* spp. are considered as the reservoir for both serovars (Ido et al., 1917; Vinetz, 1996; Ko et al., 1999; de Faria et al., 2008). Furthermore, there is no apparent difference in terms of disease outcome between *L. interrogans* Copenhageni and Icterohaemorrhagiae serovars. In spite of being serologically distinct in serotyping methods, these serovars are still considered to be genetically similar (Kmety and Dikken, 1993; Majed et al., 2005). Therefore researchers are trying to understand the phenotypical and genotypic differences between these serovars with little success.

Genotyping methods such as multilocus variable-number tandem repeat (VNTR), pulsed-field gel electrophoresis (PFGE) and multilocus sequence typing (MLST) employed to distinguish *L. interrogans* Copenhageni and Icterohaemorrhagiae serovars lack enough discriminatory power (Barocchi et al., 2001; Bharti et al., 2003; Majed et al., 2005; Galloway and Levett, 2010; Romero et al., 2011). Multispacer sequence typing (MST) represents an evolved genotyping method for the serovar identification of *L. interrogans* serogroup Icterohaemorrhagiae (Zilber et al., 2014). Though serovar variation has been related to lipopolysaccharide (LPS) structure, specifically involving the biosynthesis locus (rfb cluster) of the O-antigen, none of the above molecular typing methods are directly related to this cluster (Faine, 1994; Llanes et al., 2016). Further comparative genomic analysis of *L. interrogans* serovars Icterohaemorrhagiae and Copenhageni may reveal the differences essential for development of novel molecular serotyping techniques (Moreno et al., 2016).

DNA polymorphisms such as single nucleotide polymorphisms (SNPs), insertions and deletions (Indels), and other larger rearrangements were successfully employed to study sequence diversity among closely related but distinct populations (Gutacker et al., 2002; Joshi et al., 2012). The use of next generation sequencing (NGS) data to detect DNA polymorphisms in the context of whole-genome analysis has been previously reported in pathogenic bacteria like *Salmonella typhi, Brucella* spp. and *Bacillus anthracis* (Fournier et al., 2014). Therefore, whole-genome sequencing might serve as a robust and unbiased method to resolve intraspecies relationships in *Leptospira*.

Genome-wide identification of SNPs and Indels in *L. interrogans* serovars Copenhageni and Icterohaemorrhagiae would advance our understanding of genomic diversity of these strains isolated from various geographic locations and their evolution. Identifying the genotypic differences between both serovars can improve our understanding of their evolutionary relationships in diverse epidemiological settings. This study of genomic variations will also facilitate the development of new molecular markers to differentiate pathogenic serovars and will further aid in the leptospirosis prevention strategies. Hence we performed whole-genome sequencing of 67 different strains of *L. interrogans* serovars Copenhageni and Icterohaemorrhagiae and conducted genome-wide analyses to identify serovar-specific differences.

## Materials and methods

### *Leptospira* isolates

A total of 67 strains of *L. interrogans* serogroup Icterohaemorrhagiae, including 55 serovar Copenhageni isolates and 12 serovar Icterohaemorrhagiae isolates, were included in this study. These strains were isolated from different geographic locations and hosts, and the years of isolation ranged from 1915 to 2012 (Table S1). To include more strains for validation, eight *L. interrogans* serovar Icterohaemorrhagiae and seven serovar Copenhageni strains, whose genomes were not sequenced, were also included in this study for Sanger sequencing and/or function analyses (Table S1).

### Culture, genomic DNA extraction, and sequencing

The *Leptospira* strains were cultured in liquid Ellinghausen-McCullough-Johnson-Harris (EMJH) (Johnson and Harris, 1967) media incubated at 29°C with moderate shaking at 100 rpm. DNA was extracted from late-log phase cultures using the Maxwell 16 cell DNA purification kit along with the Maxwell DNA extraction system (Promega). The quality and concentration of DNA was measured by spectrophotometry using the NanoDrop system (Thermo Scientific, DE, USA) and by fluorometic assay using the Quanti-iT PicoGreen dsDNA assay kit (Invitrogen).

Genomes were sequenced for all 67 strains described in this study and corresponding datasets were recruited for further analyses. Genomic sequencing was done at the J. Craig Venter Institute (JCVI) using an Illumina/Solexa Genome Analyzer II, and at the Yale Center for Genome Analysis (YCGA) using the Illumina HiSeq 2000. Whole genome reads for each isolate were deposited at NCBI in the Sequence Read Archive (SRA) database (accession numbers in Table S1).

### Serological characterization of isolates

The microscopic agglutination test (MAT) was used to type *Leptospira* isolates. For serogrouping, a standard 19 panel rabbit polyclonal antisera against reference serovars representing 12 different serogroups was used, as previously described (Reis et al., 2008). For serotyping, we used different monoclonal antibodies (F89 C12-6, F70 C14, F70 C24-20, and F12 C3-11 - KIT-Biomedical Research, Amsterdam) to classify isolates of serogroup Icterohaemorrhagiae as serovar Copenhageni or serovar Icterohaemorrhagiae.

### Sequence analysis pipeline

**Figure 1:**
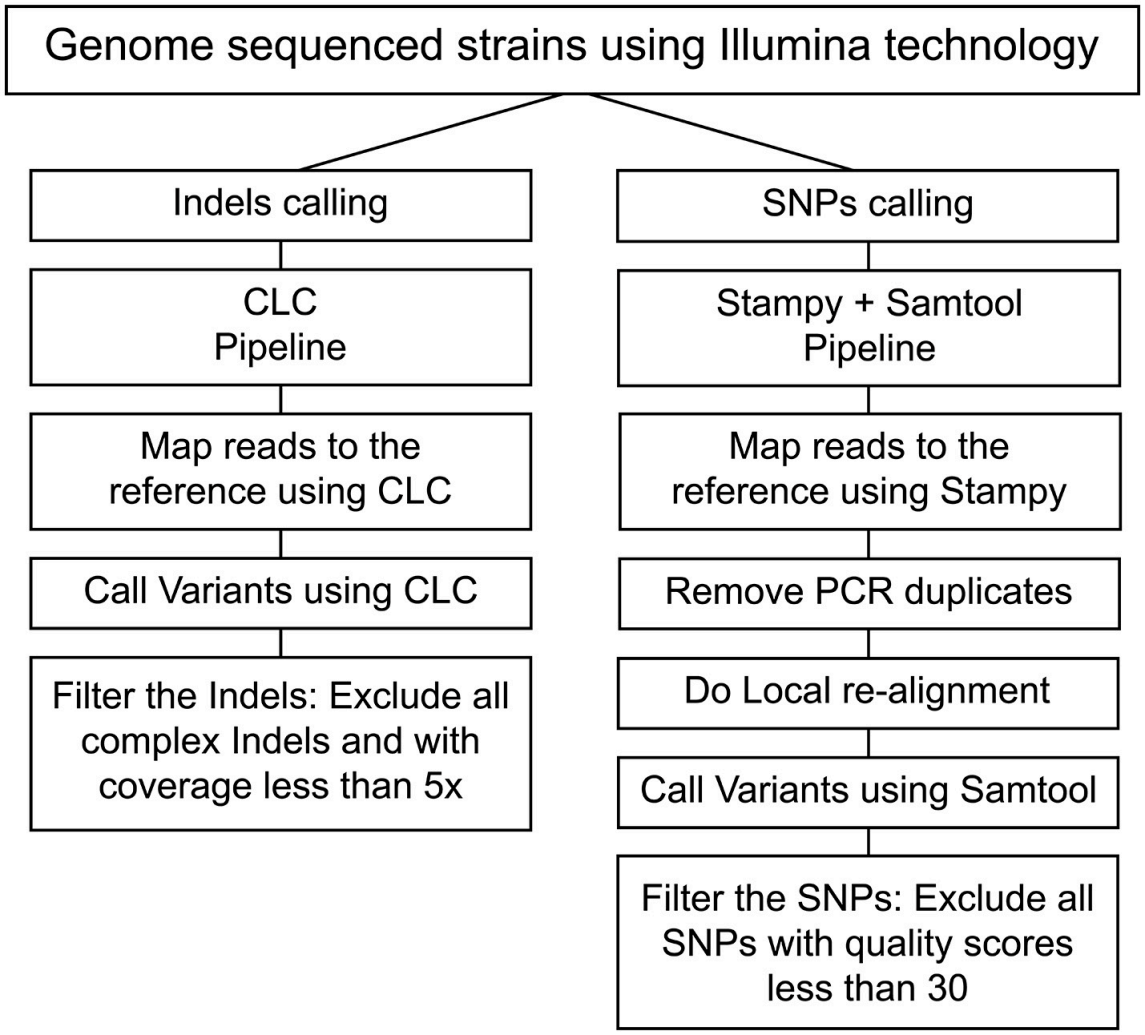
Pipeline process to identify SNPs and Indels. The flowchart represents the pipelines employed for calling SNPs (Stampy and Samtools) and Indels (CLC).

Reads were mapped to the *L. interrogans* serogroup Icterohaemorrhagiae serovar Copenhageni L1-130 strain reference genome (Nascimento et al., 2004) (Accession numbers: NC005823 and NC005824) using Stampy tool (Lunter and Goodson, 2011). The replicated alignment removal and local realignment were done using Samtools (Li et al., 2009). The processed mapping results were further analyzed for SNP calling using Samtools. Called SNPs were filtered to have a quality score cut-off >30. CLC Genomics workbench (CLC Genomics 6.0.4) was used to call Indels, and those with coverage lower than 5x were filtered. The Samtools pipeline exhibited better rates of consistency for SNPs calling while CLC was consistent for Indel calling (Figure 1, Tables S2, S3). In our reference-guided approach, we subsequently analyzed the un-mapped reads and did not detect any plasmids or misplaced contigs. To evaluate the presence of complex mutations (heterozygosis), Sanger sequencing and RFLP were performed for 3 regions. All analyses showed that the alternative allele was not present indicating a sequence error in some of the reads. Based on these analyses all the complex SNPs were excluded from this pipeline.

### Phylogenetic analyses

Phylogenetic analyses of SNPs across the whole-genome were used to infer the relationships among the 67 isolates of *L. interrogans* serogroup Icterohaemorrhagiae collected from diverse geographical locations. The reference strain *L. interrogans* serogroup Icterohaemorrhagiae serovar Copenhageni L1-130 (Nascimento et al., 2004) was included in the phylogenetic analysis. *L. interrogans* serogroup Icterohaemorrhagiae serovar Lai (Ren et al., 2003) was used as an outgroup. The length of the sequence alignment consisted of 1,731 variable sites, out of which 1072 variations were detected in this study from the sequences of serovars Copenhageni and Icterohaemorrhagiae. Additional 659 variable sites were included from the outgroup sequence serovar Lai. The Maximum Likelihood (ML) phylogeny was inferred using PAUP^*^ (Rogers and Swofford, 1998) applying the GTR model of nucleotide substitution and gamma shape parameter. Bootstrap analysis (1,000 replicates) was used to calculate the statistical support of the tree branches. Bayesian trees were also inferred including the years and country of isolation in the tree construction parameters using BEAST v.1.8 package (Drummond and Rambaut, 2007). For this dataset, we only included the 57 sequences that presented complete data from year and country of isolation (Table S1). The models tested were the strict molecular clock with constant population size prior and the relaxed molecular clock using the constant population size, Bayesian Skyline Plot (BSP), Skyride and exponential growth priors. The parameters for each model were estimated using the Monte Carlo Markov Chain (MCMC) method (50,000,000 generations with sampling every 5,000 generations). The tested models were compared calculating the Bayes Factor (BF). Using TreeAnnotator v1.4.8, included in the BEAST package (Drummond and Rambaut, 2007), the maximum clade credibility tree was summarized from the posterior tree distribution after a 50% burn-in, for each dataset (Drummond and Rambaut, 2007). MacClade was used to identify the frequency of unambiguous changes among various countries from where isolates originated in the tree (Maddison and Maddison, 1989).

### Statistical analyses

Genotypes of *L. interrogans* serogroup Icterohaemorrhagiae serovars Icterohaemorrhagiae and Copenhageni strains were compared based on the log likelihood ratio test. For a given SNP or indel, two binomial distributions were fitted for the number of alternative alleles observed separately in Icterohaemorrhagiae and Copenhageni strains by maximizing the likelihood. The supposed maximized likelihood of Icterohaemorrhagiae and Copenhageni were denoted as *L*_*I*_ and *L*_*C*_. Then another binomial distribution was fitted for the number of alternative allele by treating all strains from Icterohaemorrhagiae and Copenhageni as one group. If the maximized likelihood of this binomial distribution is *L*_*I &C*_, then the log likelihood ratio is calculated as $-2\text{log}\left(\frac{L_{I\;\&C}}{L_{I\cdot }L_{C}}\right)$. The *P* value was then calculated by comparing this observed log likelihood ratio to the Chi-squared distribution with 1 degree of freedom. To detect the degree of clustering of the genetic variation, a Principal Component Analysis (PCA) was performed using the SNPs. These analyses were performed using R.

### Identification of internal indel in *L. interrogans* serovars icterohaemorrhagiae

Sanger sequencing confirmed the observed *lic12008* mutation using gene specific primers (forward 5′TAGGTTGGCACGAAGGTTCT3′ and reverse 5′TTTTTCCGGGAACTCCAAC3′) Sequencher 5.2 (Sequencher® Gene Codes Corporation) was used to conduct the sequence analysis, and new sequences were aligned with the reference strain to identify the presence of the mutation. A total of 13 strains belonging to *L. interrogans* serovar Icterohaemorrhagiae (WGS for 5 strains) and 9 strains (WGS for 2 strains) of serovar Copenhageni were also analyzed (Table S1). BLAST analyses identified homologous sequences of LIC12008 at nucleotide and protein levels. Domain analysis of LIC12008 protein was performed using NCBI CD-search and Pfam 27.0 sequence search tools (Finn et al., 2008).

### Isolation of RNA and quantitative reverse transcription PCR (RT-qPCR)

*L. interrogans* serovar Icterohaemorrhagiae strains 201000458, 201000456 and serovar Copenhageni strain L1-130 were cultured to a density of 10^8^ bacteria per mL at 29°C with shaking. Cultures were harvested via centrifugation at 3,200 *g* and RNA was extracted for two biological replicates using the TRIzol (Invitrogen) method, as previously described. Ambion® TURBO DNA-free™ DNase Treatment kit was employed to remove contaminating DNA from RNA preparations. The concentration of RNA was determined using a Spectrophotometer (NanoDrop). The high capacity cDNA reverse transcription kit (Life Technologies) was employed for conversion of total RNA to single-stranded cDNA. Two primer sets were used to assess the impact of frameshift mutation on expression of LIC12008 (Table S7). First primer set (12008 T1) amplified a fragment of 126 bp in region encompassing nucleotides from 29 to 155 bp before the frameshift mutation. A second primer set (12008 T2) amplified a fragment of 133 bp after the mutation (from nucleotide 335–468) (Table S7).

The qPCR was carried out on 7500 fast real-time PCR (ABI, USA) using iQ™ SYBR^R^ Green supermix (Biorad) according to manufacturer's instructions. The thermal cycling conditions used in the qPCR were 95°C for 3 min, followed by 40 cycles of 95°C for 5 s and 60°C for 1 min. The specificity of the SYBR green PCR signal was confirmed by melt curve analysis. In RT-qPCR experiments, *flaB* gene was used as an endogenous control and reference strain employed was *L. interrogans* serovar Copenhageni strain L1-130. Relative quantification analysis was performed using the comparative C_t_ method, and relative gene expression was calculated by using the 2^−ΔΔ^Ct method (Schmittgen and Livak, 2008).

### *In Vivo* characterization

To test whether the *lic12008* mutation impacts virulence, *in vivo* experiments were performed in the hamster model of infection. Two strains of *L. interrogans* serovar Icterohaemorrhagiae (201000458, 201000456) were tested and compared to *L. interrogans* serovar Copenhageni strain L1-130. Groups of three 21 day old male Golden Syrian hamsters were infected with doses of 10^2^ and 10^8^ leptospires by intraperitoneal (IP) and conjunctival routes as described previously (Wunder et al., 2016).

Animals were monitored twice daily for endpoints including signs of disease and death up to 21-days post-infection. Surviving animals 21-days after infection or moribund animals at any time presenting with difficulty moving, breathing or signs of bleeding or seizure were sacrificed by CO_2_ inhalation (Wunder et al., 2016).

### Ethics statement

Animal protocols and work were approved and conducted under the guidelines of the Yale Institutional Animal Care and Use Committee (IACUC), under protocol #2017–11424. The Yale IACUC strictly adheres to all Federal and State regulations, including the Animal Welfare Act, those specified by Public Health Service, and the US Department of Agriculture and uses the *US Government Principles for the Utilization and Care of Vertebrate Animals Used in Testing, Research, and Training* as a guide for animal studies.

## Results and discussion

### Pipeline for whole-genome mapping

In this study, a total of 67 strains were sequenced, out of which 55 strains belonged to *L. interrogans* serovar Copenhageni and 12 strains belonged to *L. interrogans* serovar Icterohaemorrhagiae (Table S1). Greater number of *L. interrogans* serovar Copenhageni sequences were included in this study as this serovar is more prevalent than serovar Icterohaemorrhagiae and has greater number of isolates. All the 67 strains that were included in this study were confirmed by MAT using polyclonal sera for confirmation of Icterohaemorrhagiae serogroup (serogrouping) and monoclonal sera for differentiating Icterohaemorrhagiae and Copenhageni serovars. Serogrouping and serotyping by MAT (data not shown) confirmed the relatedness of the isolates to serogroup Icterohaemorrhagiae, and their identity to serovars Copenhageni or Icterohaemorrhagiae (Table S1). Stampy and Samtools were used for read mapping and SNP identification, respectively. Indels for both mapping and identification were analyzed using the CLC genome workbench. The pipeline used for identification of SNPs and indels was validated in this study by re-sequencing seven *Leptospira* isolates. Sequences from each of the seven isolates were analyzed and the selection of the best pipeline was made based on identification of the highest overlap percentage of SNPs and/or indels found in both sequences (Figure 1, Tables S2, S3). The information related to reads and assembly quality for these sequenced genomes can be found in Table S4.

*L. interrogans* serovar Copenhageni strain Fiocruz L1-130, sequenced using shotgun technology, was used as the reference genome (Accession numbers: NC005823 and NC005824) (Nascimento et al., 2004). Comparison of genome of *L. interrogans* serovar Copenhageni strain Fiocruz L1-130 strain re-sequenced using Illumina technology with the previously published genome sequence resulted in identification of 66 SNPs and 62 indels. Of these, 45 SNPs and 46 indels had a distribution frequency of 97% or higher in all the 132 Copenhageni strains sequenced by our group (55 included in this study and 77 from a different study) (data not shown). This higher distribution frequency of SNPs and INDELs in all the above strains might be attributed to the propagation of sequencing errors from the reference *L. interrogans* Fiocruz L1-130 sequence. In order to avoid those mutations with high frequency from the reference *L. interrogans* Fiocruz L1-130 sequence we excluded them from our analysis.

For this manuscript, we used a reference-guided approach to identify the basic differences between the genomes. However, subsequently we also employed *de novo* assembly but it did not improve the outcome. Furthermore, we checked the unmapped reads and performed a de novo assembly with those reads but this approach also did not find any misplaced contigs or reads. In our data, the assembly covered from 97.4 to 99.99% of the genome.

### Characteristics of the mutations detected in *L. interrogans* serogroup icterohaemorrhagiae serovars copenhageni and icterohaemorrhagiae strains

Whole genome sequencing enabled us to study the genome-wide variations of *L. interrogans* serovars Copenhageni and Icterohaemorrhagiae. Cumulatively, we identified 1,072 SNPs in 67 isolates, of which 276 were in non-coding region and 796 in coding regions (Table 1 and Table S5). SNPs in coding regions were distributed in 594 different genes, and 115 of those had two or more SNPs in the same gene (Table 1 and Table S5). Of the identified mutations in coding regions, 258 were synonymous and 538 were non-synonymous. The frequency of distribution of SNPs indicated a high proportion of non-synonymous to synonymous changes, at a ratio of 2:1. Previous comparative studies of closely related *B. anthracis, M. bovis*, and *Chlamydia* strains also revealed the high frequency of non-synonymous SNPs (Jordan et al., 2002; Read et al., 2002; Garnier et al., 2003). Therefore, a high proportion of non-synonymous mutations in serovars Copenhageni or Icterohaemorrhagiae could be due to their recent emergence suggesting that purifying selection might have had insufficient time to remove these slightly deleterious mutations as observed in other pathogenic bacteria (Harrison, 2013). Alternatively, this high proportion of non-synonymous mutations in both the serovars could also be an indication that some of these genes might be under positive selection.

**Table 1:** Classification and total number of identified mutations among isolates of *L. interrogans* serovars Copenhageni and Icterohaemorrhagiae.

**Mutation**	**Classification**	**Coding region**	**Non-coding region**	**Total**
SNPs	Synonymous	258	NA	258
	Non-synonymous	538	NA	538
	Total	796	276	1,072
Indels	Insertion	84	41	125
	Deletion	107	26	133
	Total	191	67	258

*NA, Not applied*.

We also identified 258 indels, of which 191 (107 deletions and 84 insertions) were found in coding regions and 67 (26 deletions and 41 insertions) in non-coding regions. Indels in coding-region were distributed in 153 different genes with 26 genes harboring two or more mutations (Table 1 and Table S6). Of the 191 indels identified in coding region 183 indels caused a frameshift in the reading frames. The resulting amino acid changes and other characteristics have been represented in Table S6. However more functional studies are required to understand if these affected genes are related to any phenotypic consequences.

Our results further identified genes with higher frequency of SNPs and indels (Table 2). However, we could not correlate this high frequency of compound mutations (multiple mutations on the same gene) with any of the phenotypic outcomes. It is possible that some of these multiple indels in the same gene could serve as compensatory indels thereby restoring the translation frame and making it a less deleterious mutation (Liu et al., 2015). Given the caveat that most of the genes in the *Leptospira* genome are annotated as hypothetical proteins or have less homology to the characterized proteins, it might be difficult to phenotypically evaluate the compensatory indels.

**Table 2:** Identification of genes showing multiple SNPs and Indels in *L. interrogans* serovars Copenhageni and Icterohaemorrhagiae.

**Mutation**	**Gene ID**	**Gene Product**	**nV^a^**	**Chr^b^**	**Gene Position^c^**	**Gene Size (bp)**
SNPs	LIC12896	Hypothetical protein	17	1	3498112	9,423
	LIC10502	Cytoplasmic protein	8	1	590325	8,364
Indels	LIC10900	Adenylate/guanylate	6	1	1085410	1,404
	LIC10674	Hypothetical protein	4	1	819364	432
	LIC13017	Acriflavin resistance	4	1	3673377	3,312

a Total number of variants in corresponding gene.

b *Chromosome*.

c *Gene position based on the L. interrogans serovar Copenhageni strain Fiocruz L1-130 reference genome (Accession numbers: NC005823 and NC005824)*.

To our knowledge, this is the first large-scale study to identify global sequence variations among *L. interrogans* Copenhageni and Icterohaemorrhagiae serovars.

### Phylogenetic analysis

**Figure 2:**
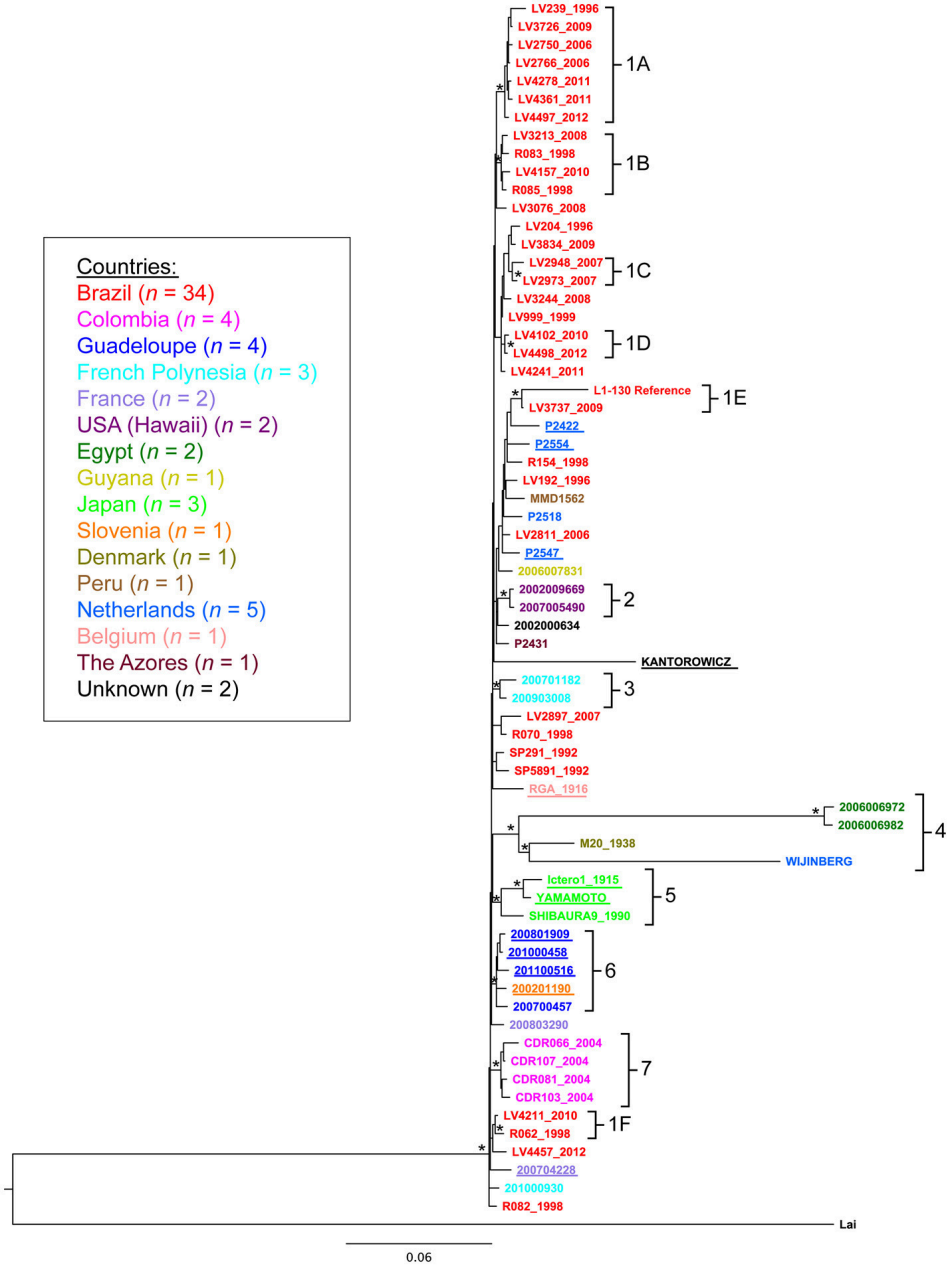
Phylogenetic relationship among *Leptospira interrogans* serovar Copenhageni and Icterohaemorrhagiae strains. Maximum likelihood tree depicting phylogenetic relationship among *Leptospira interrogans* serovars Copenhageni and Icterohaemorrhagiae from different geographical locations. Phylogeny constructed using the SNPs sites. Asterisk (^*^) represents clusters with statistical Bootstrap support higher than 70%. The sequences from serovar Icterohaemorrhagiae are underlined. *L. interrogans* serovar Lai was used as an outgroup for this analysis. The strain sequences are colored by country of isolation following the legend. The clusters numbers are related to country of isolation: 1A−1F (Brazil), 2 (Hawaii, US), 3 (French Polynesia), 4 (Egypt, Netherlands and Denmark), 5 (Japan), 6 (Guadalupe and Slovenia), and 7 (Colombia).

We constructed phylogenetic trees to gain insights into the spatial and temporal diversity of *L. interrogans* serovars Copenhageni and Icterohaemorrhagiae (Figure 2 and Figure S1). The analysis was performed with all 67 strains isolated from different geographic locations, hosts and collected during a broad range of time (Table S1). A maximum likelihood (ML) tree was constructed based on the SNPs identified in each of the 67 strains, using *L. interrogans* serogroup Icterohaemorrhagiae serovar Lai (Ren et al., 2003) as an outgroup (Figure 2). ML tree presented a topology where both serovars Copenhageni and Icterohaemorrhagiae clustered together with statistic support (Figure 2 cluster 6). In order to better interpret the internal branches and relation between genomes in Figure 2 we also constructed a Bayesian phylogenetic tree (Figure S1) without the outgroup serovar Lai. In both the trees (Figure 2 and Figure S1) we observed that Icterohaemorrhagiae and Copenhageni genomes clustered together with statistical support (represented by bootstrap for the ML tree and posterior probability for the Bayesian analyses). Therefore, our phylogenetic analyses indicated a genetic relatedness of the *L. interrogans* Copenhageni and Icterohaemorrhagiae serovars.

Despite phylogenetic relatedness, clones of strains from the Icterohaemorrhagiae serogroup seem to have evolved and adapted in different locations. Phylogenetic analysis showed seven geographic clades that corresponded spatially with the potential origins of the isolates (Table 3, Figure 2). The bootstrap support of higher than 70% was employed based on the general consensus (Hillis and Bull, 1993) and statistical support for these clades was further confirmed by principal components analysis (PCA) (data not shown). These clades include isolates from Brazil (clade 1A to 1F), Hawaii (USA, clade 2), French Polynesia (clade 3), Egypt, Denmark, Netherlands (clade 4), Japan (clade 5), Guadalupe and Slovenia (clade 6) and Colombia (clade 7) (Table 3). To substantiate our results for geographical clustering, Bayesian analysis was performed for 57 isolates of *L. interrogans* serovars Copenhageni and Icterohaemorrhagiae, which had specific information related to their location and year of isolation. The temporal and spatial information was incorporated into this analysis and a relaxed clock with Bayesian skyline plot was selected as the best explanatory model. The resulting tree for spatiotemporal analyses was similar to the ML tree, with distinct geographic clades from Brazil, Colombia, Guadalupe, French Polynesia, Hawaii, Egypt, and Japan (Figure S1).

**Table 3:** Correlation of isolates based on the geographical clades from phylogenetic analysis.

**Clade**	**Isolate^a^**	**Country^b^**
1A	LV239, LV3726, LV2750, LV2766, LV4278, LV4361, LV4497	Brazil
1B	LV3213, R83, LV4157, R85	Brazil
1C	LV2948, LV2973	Brazil
1D	LV4102, LV4498	Brazil
1E	L1-130, LV3737	Brazil
1F	LV4211, R62	Brazil
2	2007005490, 2002009669	Hawaii
3	200903008, 200701182	French Polynesia
4	2006006972, 200600682, M20, Wijnberg	Egypt, Denmark, Netherlands
5	Ictero1, Yamamoto, Shibaura	Japan
6	200801909, 201000458, 201100516, 200700457, 200201190	Guadeloupe, Slovenia
7	CIDEIM R006, CIDEIM R107, CIDEIM R081, CIDEIM R103	Colombia

a,b *Details of all the isolates and countries of origin are listed in Table S1*.

Isolates of *L. interrogans* serovar Copenhageni from Salvador, Brazil, were significantly represented in both phylogenies and exhibited a mixed clustering pattern with isolates from other countries (Figure 2). The frequency of unambiguous changes between states over the maximum parsimonious trees (MPTs) was estimated in MacClade (Figure S2). Significant distribution of *L. interrogans* serovars was observed between European Union to Egypt (25%) and Guadeloupe to Slovenia (12.5%). Other small distribution events observed were from Brazil to Guyana and French Polynesia (1.5%). One of our important interpretations arising from the MacClade analysis was that strains from Egypt could have ancestral origins in Europe (Figure S2). Taken together, the MacClade analyses indicated few instances of distribution events within *L. interrogans* serovars across the globe and particularly in Salvador, Brazil.

Human connectivity with infectious rodent host reservoirs (*R. novergicus*) via transcontinental trading by mercantile ships from Europe, Africa and other continents is one plausible explanation for the introduction of various strains of *L. interrogans* serovar Copenhageni strains into Salvador. A recent study explored the global population structure of *R. novergicus* and suggested that brown rats expanded across Asia, Europe and North America through human settlements associated with Silk Road trade routes (Puckett et al., 2016). Therefore, it is possible that strains of *L. interrogans* serogroup Icterohaemorrhagiae might have been spread via rodent host reservoir. However, high level of sequence similarity among serogroup Icterohaemorrhagiae isolates indicates that specific SNPs/Indels observed were essential for their survival and prevalence in diverse habitats.

### Identification of a unique indel in serovar icterohaemorrhagiae strains

**Figure 3:**
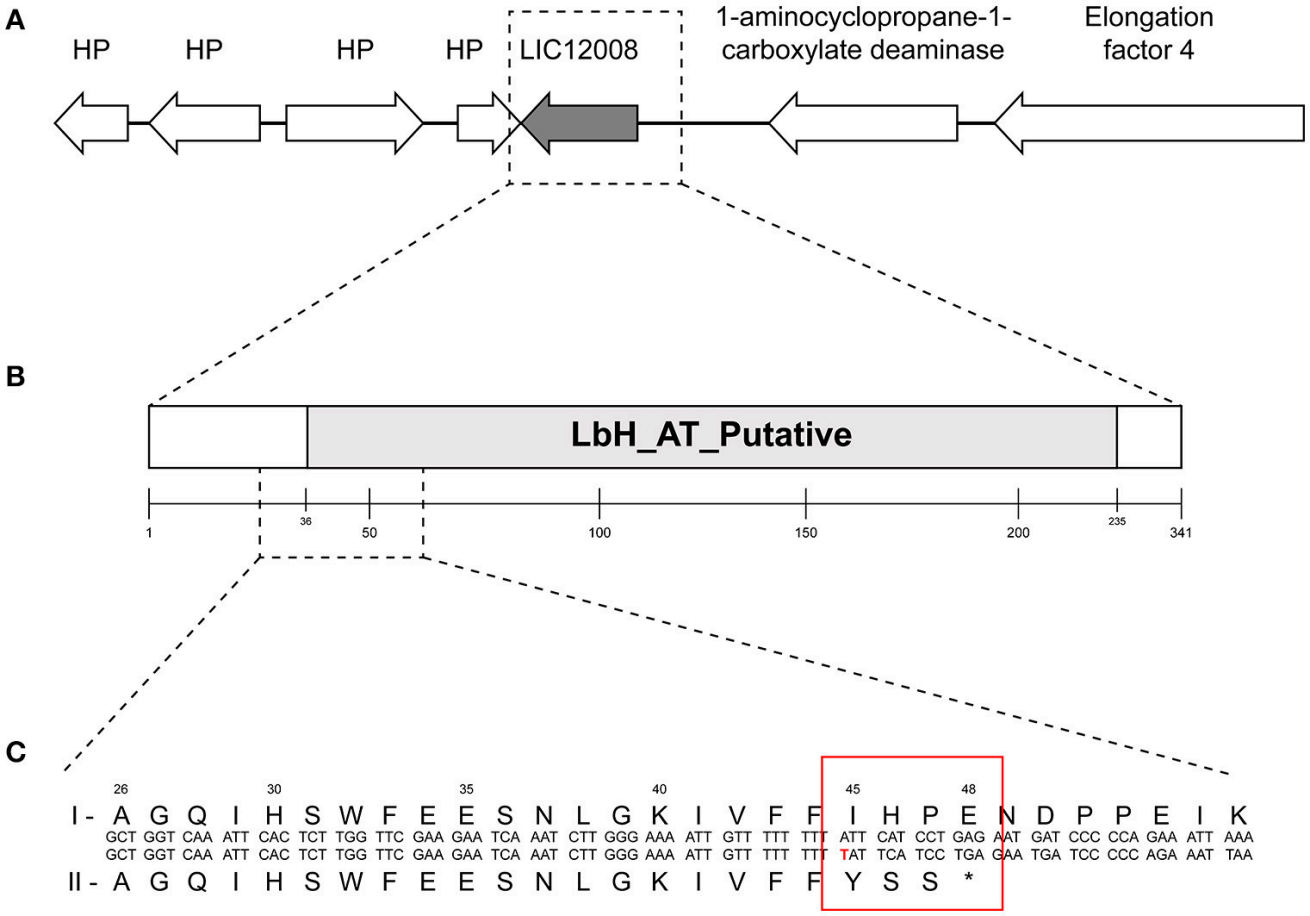
Genomic organization and domain architecture of gene *lic12008*. **(A)** Organization of *lic12008* loci in *L. interrogans* serovars. **(B)** Domain architecture of LIC12008 protein. **(C)** Details of amino acid fragment (1-55aa) showing the sequence differences in *L. interrogans* Copenhageni (I) and *L. interrogans* Icterohaemorrhagiae (II) serovars.

To identify the genetic mutation that could differentiate both serovars, a likelihood ratio test (LRT) of the SNP analyses was performed. LRT suggests that no SNPs distinguished these two serovars of **Icterohaemorrhagiae and Copenhageni**. However, LRT of the indel data was statistically consistent (*p* = 0.039) with the presence of a single base insertion of a thymine nucleotide within a polyT tract (9 bp long) in gene *lic12008* (Figures 3A,B) in all the *L. interrogans* serovar Icterohaemorrhagiae isolates. As an orthogonal approach, we ran parsimony informative test using our SNPs and it yielded 30% of parsimony-informative sites (data not shown). However, except for the one indel in *lic12008* locus we did not identify any statistically significant SNP's in our analyses.

The gene *lic12008* (726 bp) is located at nucleotide positions 2416786 to 2417511 of Chromosome 1 (reading frame of −2) in *L. interrogans* serovar Copenhageni Fiocruz L1-130 genome and was not found to be part of any operon. Sanger sequencing confirmed the presence of this insertion at the 5′ end of *lic12008* gene in all *L. interrogans* Icterohaemorrhagiae strains but its absence in Copenhageni strains (data not shown). The identified insertion in *lic12008* gene resulted in a frameshift mutation at N terminal region (45th amino acid) of the corresponding protein (Figure 3C). This highly conserved change at amino acid level within the serovar Icterohaemorrhagiae might have evolutionary implications leading to its divergence from the Copenhageni serovar. A significant overrepresentation of frameshifts at N terminal region of proteins was also previously observed in pathogenic *P. aeruginosa* (Harrison, 2013).

Our results indicated that an internal indel in a homopolymeric tract region explains one key aspect of sequence diversity between closely related *L. interrogans* serovars Copenhageni and Icterohaemorrhagiae strains. This observation agrees with previous results where homopolymeric regions were known to be subject to indel mutations as major sources of sequence diversity in various animal, plants, insects and bacteria (Moran et al., 2009). However, indels in homopolymers more often have deleterious effects, and there is an increasing evidence for strong purifying selection against frameshift mutations in coding regions (Williams and Wernegreen, 2013). Therefore, the conserved frameshifting indel in *lic12008* observed in *L. interrogans* serovar Icterohaemorrhagiae isolates is interesting from an evolutionary perspective.

In this study, the power of variant discovery was enhanced by jointly analyzing all the samples (Li and Durbin, 2010; DePristo et al., 2011; Nielsen et al., 2011). Subsequently genotype likelihood-based LRT was assessed to compute a statistically robust association that was able to classify *L. interrogans* serovars Copenhageni and Icterohaemorrhagiae isolates. This test combined with the number of sequences analyzed, demonstrated that both serovars are highly related beyond serovar classification. Thus, the majority of SNPs and indels detected among the 67 strains represented the natural diversity of the sequences. In a recent study, MST was employed to differentiate Copenhageni and Icterohaemorrhagiae serovars. However, MST could not define unique profiles for few strains of *L. interrogans* serovars Copenhageni (M20 and Wijnberg strains) and Icterohaemorrhagiae (RGA and Verdun strains) (Zilber et al., 2014). The identified Indel in *lic12008* gene however had a high discriminatory power to distinguish between Copenhageni and Icterohaemorrhagiae serovars without any exceptions.

### Analysis of genomic region with predicted indels

Basic local alignment search tool (BLAST) was used to study the distribution of *lic12008* gene in other *Leptospira* species. Nucleotide analyses demonstrated that this gene was absent in non-pathogenic and intermediate *Leptospira* species and present only in four pathogenic species: *L. interrogans, L. kirschneri, L. noguchii*, and *L. santarosai*. BLAST with the LIC12008 amino acid sequence as query (cutoff: >30% identity) identified UDP-3-O-(3-hydroxymyristoyl) glucosamine N-acyltransferase, sugar O-acyltransferase and sialic acid O-acetyltransferase of NeuD family as closest homologs. In other bacteria, UDP-3-O-(3-hydroxymyristoyl) glucosamine N-acyltransferase is involved in the biosynthesis of lipid A, a phosphorylated glycolipid that anchors the lipopolysaccharide to the outer membrane of the cell (Bartling and Raetz, 2009). Previous studies demonstrated the physiological relevance of *lpxD1* gene (LIC13046) encoding a UDP-3-O-(3-hydroxymyristoyl) glucosamine N-acyltransferase in *L. interrogans* serovar Manilae strain L495 (Eshghi et al., 2015).

We identified a paralog of UDP-3-O-(3-hydroxymyristoyl) glucosamine N-acyltransferase, annotated as an acetyl transferase gene (LIC12184), in *Leptospira interrogans* Copenhageni Fiocruz L1-130, employing LIC12008 as query sequence (with identity of 74% and similarity of 89%). Further genomic characterization of this paralog might unveil the evolutionary mechanisms underlying the development of this gene family.

Domain analysis of the hypothetical protein encoded by *lic12008* showed that the region spanning amino acids 36 to 233 (out of 242aa) is comprised of the putative Acyltransferase (AT) and Left-handed parallel beta-Helix (LbH) domain (*E*-value: 6.33e-48 and domain accession cd03360) (Figure 3B). LIC12008 belongs to LbetaH superfamily proteins composed mainly of acyltransferases [33]. Three imperfect tandem repeats of a hexapeptide repeat motif (X-[STAV]-X-[LIV]-[GAED]-X) were also identified in LIC12008. Thus, the presence of LbHAT domain in LIC12008 (Figure 3B) allows us to speculate that this protein has a role in LPS biosynthesis. Previous studies implicated the role of horizontal transfer of genes located within the *rfb* cluster for serological relatedness of genetically similar serovars (de la Peña-Moctezuma et al., 1999; Nalam et al., 2010). In contrast our study identified an indel in *lic12008* gene unrelated to *rfb* cluster but with a presumable role in LPS biosynthesis. Further gene neighborhood analysis of *lic12008* did not show any evidence of horizontal gene transfer events (data not shown).

In clinical strains of *Burkholderia pseudomallei*, accumulation of four indels affecting lipopolysaccharide (LPS) biosynthesis was identified as a mechanism used by this pathogen to evade the immune response (Price et al., 2013). In this context, the presence of a frameshifting indel in *lic12008*, a LPS biosynthesis related gene seems important. Since protective antibody responses for *Leptospira* are against LPS, altered expression of LPS might have an impact on the host immune response, which might provide a plausible explanation for the serological differences found between Copenhageni and Icterohaemorrhagiae strains.

### Functional analysis

Gene expression analysis was performed to identify differences in expression of the *lic12008* transcript in *L. interrogans* serovars Copenhageni and Icterohaemorrhagiae. Two pairs of primers were used to study the impact of mutation on *lic12008* expression. The first primer (12008 T1) encompassed the region of the mutation while the second primer was outside the mutation (12008 T2) (Figure 3C, Table S7). Transcripts from both regions of *L. interrogans* serovar Icterohaemorrhagiae were downregulated compared to *L. interrogans* Copenhageni Fiocruz L1-130 (Figure S3).

To determine the possible phenotypic consequences of the *lic12008* frameshifting indel in *L. interrogans* serovar Icterohaemorrhagiae, we performed an *in vivo* experiment in hamster model of infection. Representative strains from *L. interrogans* serovar Icterohaemorrhagiae were used to infect two groups of three hamsters, via intraperitoneal and conjunctival routes respectively. Both strains of serovar Icterohaemorrhagiae were virulent in the hamster model of infection (Table S8) similar to Copenhageni strains which were previously shown to be virulent (Silva et al., 2008). This indicates that *lic12008* frameshifting indel might not be associated with any negative effects on virulence. Alternatively, paralogous gene of *lic12008* might compensate for any functional aberrations caused by this frameshifting indel.

In summary, we analyzed the genome-wide SNPs and indels among the *L. interrogans* serovars Copenhageni and Icterohaemorrhagiae isolates. Analyses of these genome-wide variations revealed that both serovars are genetically similar. Phylogenetic analyses also indicated that *L. interrogans* serovar Copenhageni and Icterohaemorrhagiae strains are highly conserved along time with a distinct geographical clustering. However, our results indicated that an internal indel is the major source of variation in *L. interrogans* serovar Icterohaemorrhagiae. This identified internal indel could provide a plausible explanation for the unexplained antigenic differences between *Leptospira interrogans* serovars Icterohaemorrhagiae and Copenhageni. Previously, indel studies in other species have led to the identification of powerful taxon diagnostics and phylogenetic markers (Baldauf and Palmer, 1993; de Jong et al., 2003; Ajawatanawong and Baldauf, 2013). In this context, the internal indel identified in this study could be possibly validated as a diagnostic marker to differentiate *L. interrogans* Copenhageni and Icterohaemorrhagiae isolates.

## Author contributions

LS, AK, EW, and MR designed research. LS and HA performed sample preparation and total DNA extraction. DF and JV performed the sequencing of the isolates. LS and XY analyzed the sequencing data. XY and HZ performed the statistical analyses. LS, LA, and JT performed the evolutionary analyses. HA performed the RT-qPCR experiment and functional analyses. LS and HA drafted the manuscript and also revised the draft. All authors read and approved the final manuscript.

### Conflict of interest statement

The authors declare that the research was conducted in the absence of any commercial or financial relationships that could be construed as a potential conflict of interest.

## Supplementary material

Figure S1 Bayesian maximum clade credibility phylogenetic tree. The tree was constructed using the relaxed molecular clock model and Bayesian Skyline Plot population size model. The branch support is given by posterior probability >0.88 and is indicated with an asterisk (^*^). The tree branches are colored by country.

Figure S2 MacClade analysis. Identification of the migration relation between strains from different geographic locations. The selected regions were Brazil (BR), United States (US), Slovenia (SL), Egypt (EG) Guyana (GY), Guadalupe (GP), European Union (EU), French Polynesia (FP), Colombia (CO), and Japan (JP).

Figure S3 Gene expression analysis of *lic12008* in *L. interrogans* serovars. Real-time PCR analysis of *lic12008* transcript in *L. interrogans* serovar Icterohaemorrhagiae strains (201000456 and 201000458) by employing primer pairs (12008 T1 and T2). Fold change was calculated by using the 2^−ΔΔCt^ method. Data was normalized by the amount of *flaB* transcript expressed relative to the corresponding value for *L. interrogans* serovar Copenhageni L1-130 and is expressed as mean ± SD.

Table S1 General information list of all the *Leptospira interrogans* strains of the serogroup Icterohaemorrhagiae used in this study.

Table S2 Validation rate for the pipeline call for SNPs.

Table S3 Validation rate for the pipeline call for Indel.

Table S4 Sequencing and mapping quality.

Table S5 List of polymorphic nucleotide sites identified in *L. interrogans* serovars from this study.

Table S6 List of Insertion and deletions (Indels) identified in *L. interrogans* serovars from this study.

Table S7 Primers employed for qRT-PCR.

Table S8 *In vivo* testing of virulence for *L. interrogans* serovar Icterohaemorrhagiae strains.
