# Supplementary figures and images for: Genomic Comparison Among Global Isolates of L. interrogans Serovars Copenhageni and Icterohaemorrhagiae Identified Natural Genetic Variation Caused by an Indel
Source: Front Cell Infect Microbiol. 2018 Jun 19;8:193. doi: 10.3389/fcimb.2018.00193 (PMC6018220; doi:10.3389/fcimb.2018.00193)

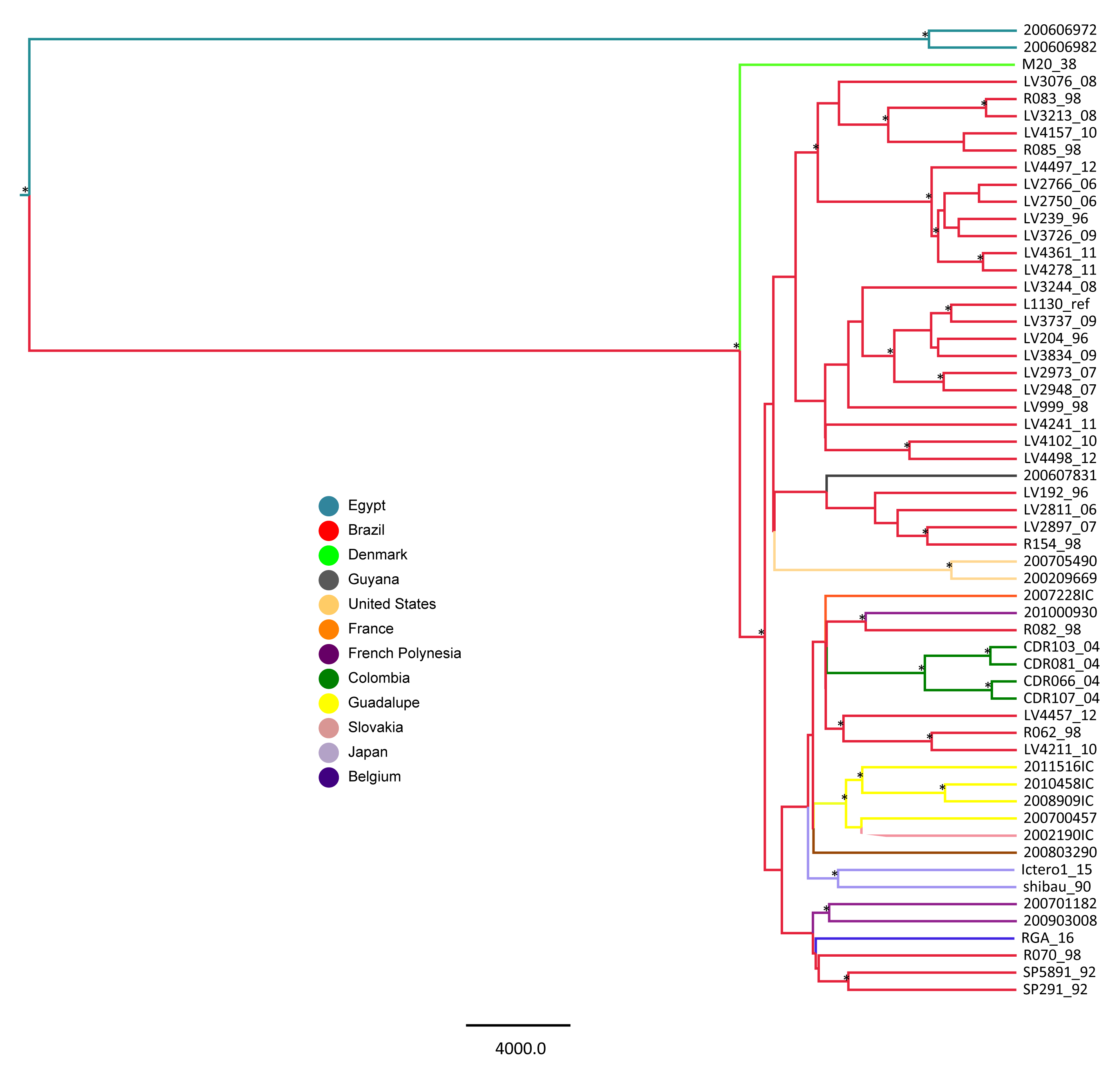

Supplement: Figure S1 — Bayesian maximum clade credibility phylogenetic tree. The tree was constructed using the relaxed molecular clock model and Bayesian Skyline Plot population size model. The branch support is given by posterior probability >0.88 and is indicated with an asterisk (*). The tree branches are colored by country. [file Image_1.JPEG]

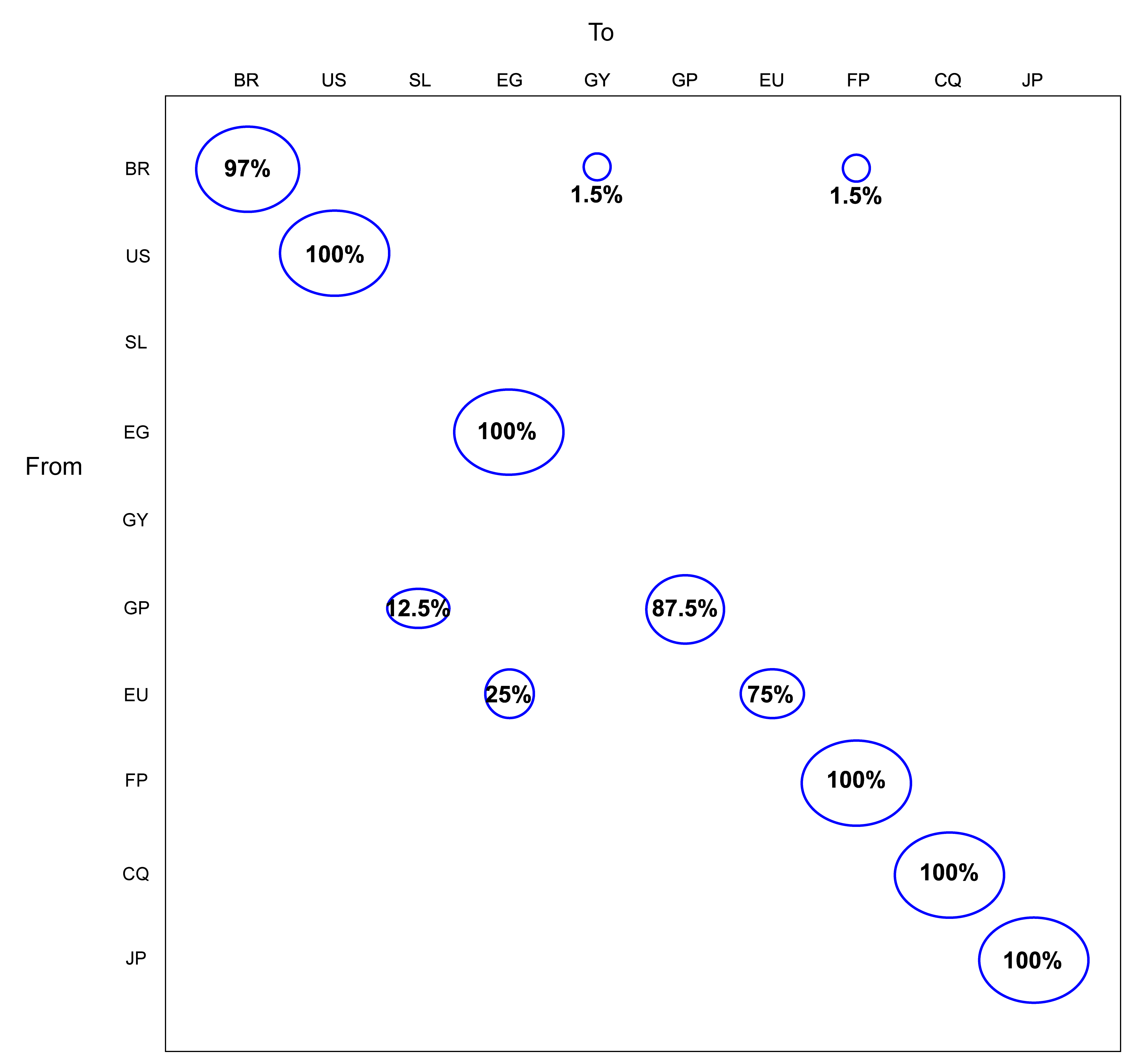

Supplement: Figure S2 — MacClade analysis. Identification of the migration relation between strains from different geographic locations. The selected regions were Brazil (BR), United States (US), Slovenia (SL), Egypt (EG) Guyana (GY), Guadalupe (GP), European Union (EU), French Polynesia (FP), Colombia (CO), and Japan (JP). [file Image_2.JPEG]

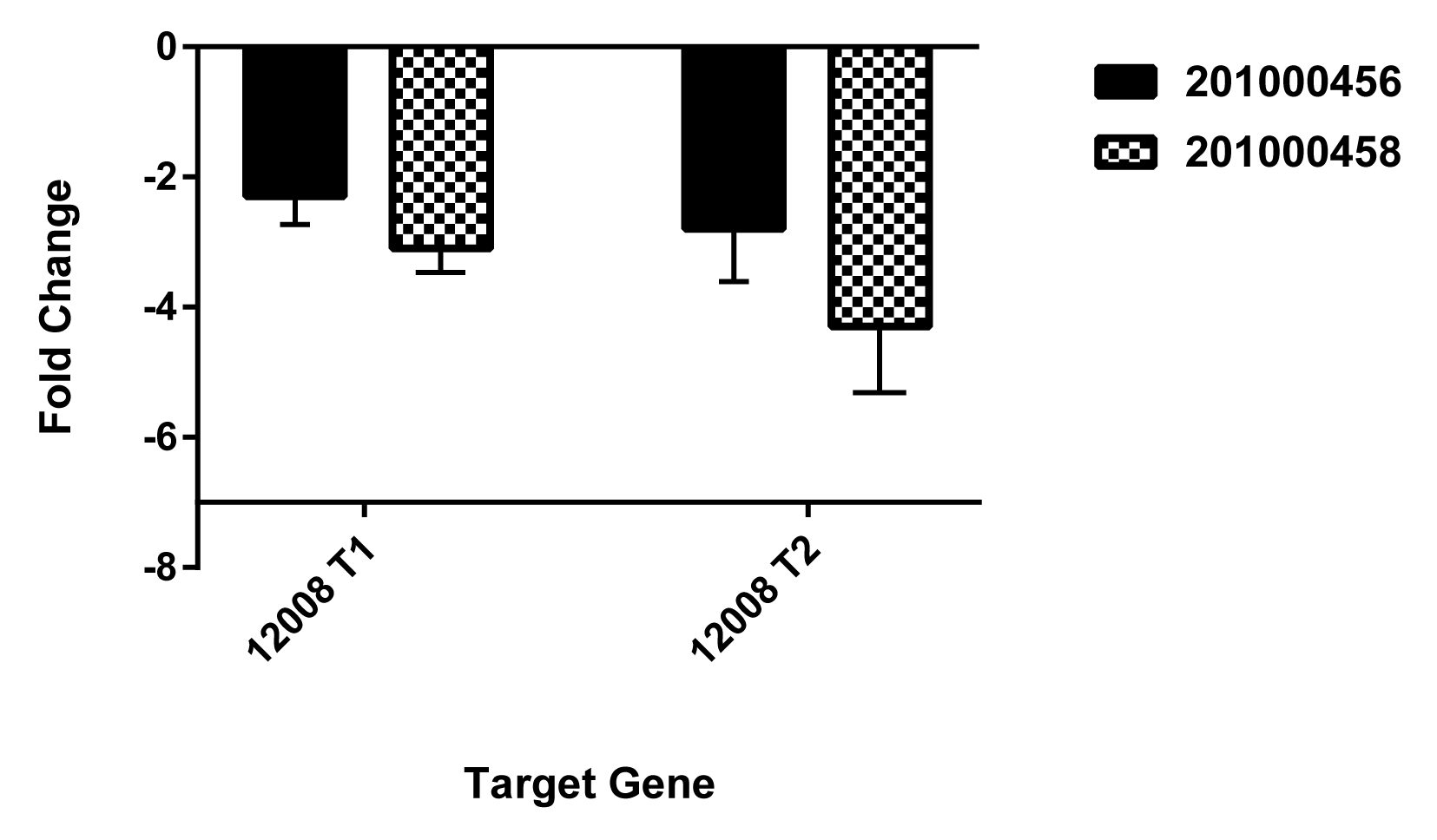

Supplement: Figure S3 — Gene expression analysis of lic12008 in L. interrogans serovars. Real-time PCR analysis of lic12008 transcript in L. interrogans serovar Icterohaemorrhagiae strains (201000456 and 201000458) by employing primer pairs (12008 T1 and T2). Fold change was calculated by using the 2−ΔΔCt method. Data was normalized by the amount of flaB transcript expressed relative to the corresponding value for L. interrogans serovar Copenhageni L1-130 and is expressed as mean ± SD. [file Image_3.TIFF]
