# Supplementary material for: Genomic Comparison Among Global Isolates of L. interrogans Serovars Copenhageni and Icterohaemorrhagiae Identified Natural Genetic Variation Caused by an Indel
Source: Front Cell Infect Microbiol. 2018 Jun 19;8:193. doi: 10.3389/fcimb.2018.00193 (PMC6018220; doi:10.3389/fcimb.2018.00193)
Supplement: Table S1 — General information list of all the Leptospira interrogans strains of the serogroup Icterohaemorrhagiae used in this study. [file Table_1.DOCX]

**Table S1.** General information list of all the *Leptospira interrogans* strains of the serogroup Icterohaemorrhagiae used in this study

| **Serovar** | **Strain** | **Host** | **Country** | **Year** | **Genome Accession number** | **LIC12008 Sanger sequencing** | **LIC12008 Gene expression analysis** | **Virulence analysis** |
| --- | --- | --- | --- | --- | --- | --- | --- | --- |
| Copenhageni | CIDEIM R066 | Rat | Colombia | 2004 | SRX055261 | - | - | - |
| Copenhageni | CIDEIM R081 | Rat | Colombia | 2004 | SRX1295494 | - | - | - |
| Copenhageni | CIDEIM R103 | Rat | Colombia | 2004 | SRX237045 | - | - | - |
| Copenhageni | CIDEIM R107 | Rat | Colombia | 2004 | SRX1295495 | - | - | - |
| Copenhageni | Fiocruz LV192 | Human | Brazil | 1996 | SRX055276 | - | - | - |
| Copenhageni | Fiocruz LV204 | Human | Brazil | 1996 | SRX055274 | - | - | - |
| Copenhageni | Fiocruz LV239 | Human | Brazil | 1996 | SRX055281 | - | - | - |
| Copenhageni | Fiocruz LV2750 | Human | Brazil | 2006 | SRX237194 | - | - | - |
| Copenhageni | Fiocruz LV2766 | Human | Brazil | 2006 | SRR715767 | - | - | - |
| Copenhageni | Fiocruz LV2811 | Human | Brazil | 2006 | SRX055230 | - | - | - |
| Copenhageni | Fiocruz LV2897 | Human | Brazil | 2007 | SRX055235 | - | - | - |
| Copenhageni | Fiocruz LV2948 | Human | Brazil | 2007 | SRX055240 | - | - | - |
| Copenhageni | Fiocruz LV2973 | Human | Brazil | 2007 | SRX237225 | - | - | - |
| Copenhageni | Fiocruz LV3076 | Human | Brazil | 2008 | SRR712964 | - | - | - |
| Copenhageni | Fiocruz LV3213 | Human | Brazil | 2008 | SRR717628 | - | - | - |
| Copenhageni | Fiocruz LV3244 | Human | Brazil | 2008 | SRX236775 | - | - | - |
| Copenhageni | Fiocruz LV3726 | Human | Brazil | 2009 | SRX055253 | - | - | - |
| Copenhageni | Fiocruz LV3737 | Human | Brazil | 2009 | SRX055255 | - | - | - |
| Copenhageni | Fiocruz LV3834 | Human | Brazil | 2009 | SRX055256 | - | - | - |
| Copenhageni | Fiocruz LV4102 | Human | Brazil | 2010 | SRX1156805 | - | - | - |
| Copenhageni | Fiocruz LV4157 | Human | Brazil | 2010 | SRX1156808 | - | - | - |
| Copenhageni | Fiocruz LV4211 | Human | Brazil | 2010 | SRX236694 | - | - | - |
| Copenhageni | Fiocruz LV4241 | Human | Brazil | 2011 | SRX1156979 | - | - | - |
| Copenhageni | Fiocruz LV4278 | Human | Brazil | 2011 | SRX1156981 | - | - | - |
| Copenhageni | Fiocruz LV4361 | Human | Brazil | 2011 | SRX1160351 | - | - | - |
| Copenhageni | Fiocruz LV4457 | Human | Brazil | 2012 | SRX1160352 | - | - | - |
| Copenhageni | Fiocruz LV4497 | Human | Brazil | 2012 | SRX1160353 | - | - | - |
| Copenhageni | Fiocruz LV4498 | Human | Brazil | 2012 | SRX1162756 | - | - | - |
| Copenhageni | Fiocruz LV999 | Human | Brazil | 1998 | SRX237193 | - | - | - |
| Copenhageni | Fiocruz R062 | Rat | Brazil | 1998 | SRX1274269 | - | - | - |
| Copenhageni | Fiocruz R070 | Rat | Brazil | 1998 | SRX1274569 | - | - | - |
| Copenhageni | Fiocruz R082 | Rat | Brazil | 1998 | SRX1274570 | - | - | - |
| Copenhageni | Fiocruz R083 | Rat | Brazil | 1998 | SRX055259 | - | - | - |
| Copenhageni | Fiocruz R085 | Rat | Brazil | 1998 | SRX1295493 | - | - | - |
| Copenhageni | Fiocruz R154 | Rat | Brazil | 1998 | SRX237044 | - | - | - |
| Copenhageni | Fiocruz LV3985 | Human | Brazil | 2010 | - | X | - | - |
| Copenhageni | Fiocruz LV4270 | Human | Brazil | 2011 | - | X | - | - |
| Copenhageni | Fiocruz LV4273 | Human | Brazil | 2011 | - | X | - | - |
| Copenhageni | Fiocruz LV4294 | Human | Brazil | 2011 | - | X | - | - |
| Copenhageni | Fiocruz LV4535 | Human | Brazil | 2012 | - | X | - | - |
| Copenhageni | M20 | Human | Denmark | 1938 | SRX1305354 | - | - | - |
| Copenhageni | MMD1562 | Bat | Peru | ND | SRX236211 | - | - | - |
| Copenhageni | P2431 | Human | Azores Island | ND | SRX236906 | - | - | - |
| Copenhageni | P2518 | Human | Netherlands | ND | SRX101365 | - | - | - |
| Copenhageni | Wijnberg | Human | Netherlands | ND | SRX236777 | - | - | - |
| Copenhageni | SP 2/91 | NA | Brazil | 1992 | SRX3235897 | - | - | - |
| Copenhageni | SP 58/91 | NA | Brazil | 1992 | SRR2559276 | - | - | - |
| Copenhageni | Fiocruz LV130 3.7 | Human | Brazil | 1996 | SRR2634502 | - | - | - |
| Copenhageni | Shibaura 9 (Yanagawa) | Human | Japan | 1990 | SRX1305359 | - | - | - |
| Copenhageni | 200700457 | Human | Guadeloupe | 2007 | SRX1305358 | X | - | - |
| Copenhageni | 200701182 | Human | French Polynesia | 2007 | SRX236905 | - | - | - |
| Copenhageni | 200803290 | Human | France | 2008 | SRX1305355 | - | - | - |
| Copenhageni | 200903008 | Human | French Polynesia | 2009 | SRX1305356 | X | - | - |
| Copenhageni | 201000929 | Human | French Polynesia | 2010 | - | X | - | - |
| Copenhageni | 201000930 | Human | French Polynesia | 2010 | SRX1305357 | - | - | - |
| Copenhageni | 201001055 | Human | Guadeloupe | 2010 | - | X | - | - |
| Copenhageni | 2002000634 | Human | ND | 2002 | SRR715898 | - | - | - |
| Copenhageni | 2002009669 | Human | Hawaii | 2002 | SRR717749 | - | - | - |
| Copenhageni | 2006006972 | Human | Egypt | 2006 | SRR717872 | - | - | - |
| Copenhageni | 2006006982 | Human | Egypt | 2006 | SRR714175 | - | - | - |
| Copenhageni | 2006007831 | Human | Guyana | 2006 | SRR171626 | - | - | - |
| Copenhageni | 2007005490 | Human | Hawaii | 2007 | SRR717631 | - | - | - |
| Icterohaemorrhagiae | Ictero1 | Guinea-Pig | Japan | 1915 | SRX1383975 | X | - | - |
| Icterohaemorrhagiae | Kantorowicz | ND | ND | ND | SRR715899 | - | - | - |
| Icterohaemorrhagiae | P1879 | Human | Netherlands | 1988 | - | X | - | - |
| Icterohaemorrhagiae | P2422 | Human | Netherlands | ND | SRX101371 | - | - | - |
| Icterohaemorrhagiae | P2547 | Human | Netherlands | ND | SRX101367 | - | - | - |
| Icterohaemorrhagiae | P2554 | Human | Netherlands | ND | SRX101366 | - | - | - |
| Icterohaemorrhagiae | P2584 | Human | Netherlands | 2009 | - | X | - | - |
| Icterohaemorrhagiae | P2628 | Human | Netherlands | 2010 | - | X | - | - |
| Icterohaemorrhagiae | RGA | Human | Belgium | 1916 | SRR715768 | - | - | - |
| Icterohaemorrhagiae | Rijinsburger | ND | ND | 1995 | - | X | - | - |
| Icterohaemorrhagiae | 1 (Yamamoto) | Human | Japan | ND | SRX1250682 | X | - | - |
| Icterohaemorrhagiae | UKKEL 54 | ND | Belgium | 1989 | - | X | - | - |
| Icterohaemorrhagiae | 200201190 | Human | Slovenia | 2002 | SRX1250683 | X | - | - |
| Icterohaemorrhagiae | 200701041 | Human | French Polynesia | 2007 | - | X | - | - |
| Icterohaemorrhagiae | 200702614 | Human | France | 2007 | - | X | - | - |
| Icterohaemorrhagiae | 200704228 | Human | France | 2007 | SRX1250684 | X | - | - |
| Icterohaemorrhagiae | 200801909 | Human | Guadeloupe | 2008 | SRX1250685 | - | - | - |
| Icterohaemorrhagiae | 201000456 | Human | Guadeloupe | 2010 | - | X | X | X |
| Icterohaemorrhagiae | 201000458 | Human | Guadeloupe | 2010 | SRX1250686 | X | X | X |
| Icterohaemorrhagiae | 201100516 | Human | Guadeloupe | 2011 | SRR714873 | - | - | - |

- = Not determined

X= Strains tested
