# Supplementary material for: Genomic Comparison Among Global Isolates of L. interrogans Serovars Copenhageni and Icterohaemorrhagiae Identified Natural Genetic Variation Caused by an Indel
Source: Front Cell Infect Microbiol. 2018 Jun 19;8:193. doi: 10.3389/fcimb.2018.00193 (PMC6018220; doi:10.3389/fcimb.2018.00193)
Supplement: Table S2 — Validation rate for the pipeline call for SNPs. [file Table_2.DOCX]

**Table S2.** Validation rate for the pipeline call for SNPs

| Strain | CLC | | | |  | Stampy + Samtools | | |
| --- | --- | --- | --- | --- | --- | --- | --- | --- |
|  | No. of SNPs detected in the first sequence | No. of SNPs detected in the second sequence | | CLC validation rate |  | No. of SNPs detected in the first sequence | No. of SNPs detected in the second sequence | Samtools validation rate |
| CIDEIM103 | 102 | 81 | 0.77 | |  | 91 | 95 | 1.00 |
| LV2776 | 84 | 63 | 0.74 | |  | 73 | 75 | 1.00 |
| LV2791 | 93 | 73 | 0.77 | |  | 77 | 83 | 0.99 |
| LV2805 | 78 | 62 | 0.76 | |  | 62 | 71 | 0.98 |
| LV2953 | 88 | 77 | 0.78 | |  | 76 | 87 | 0.97 |
| LV3094 | 87 | 70 | 0.78 | |  | 71 | 84 | 0.97 |
| LV212 | 72 | 59 | 0.72 | |  | 59 | 71 | 0.98 |

No. : = Number
