# Supplementary material for: Genomic Comparison Among Global Isolates of L. interrogans Serovars Copenhageni and Icterohaemorrhagiae Identified Natural Genetic Variation Caused by an Indel
Source: Front Cell Infect Microbiol. 2018 Jun 19;8:193. doi: 10.3389/fcimb.2018.00193 (PMC6018220; doi:10.3389/fcimb.2018.00193)
Supplement: Table S3 — Validation rate for the pipeline call for Indel. [file Table_3.DOCX]

**Table S3.** Validation rate for the pipeline call for Indel

| Strain | CLC | | |  | Stampy+Samtools | | |
| --- | --- | --- | --- | --- | --- | --- | --- |
|  | No. of Indels detected in the first sequence | No. of Indels detected in the second sequence | CLC validation rate |  | No. of Indels detected in the first sequence | No. of Indels detected in the second sequence | Samtools validation rate |
| CIDEIM103 | 72 | 68 | 0.93 |  | 90 | 95 | 0.81 |
| LV2776 | 68 | 61 | 0.90 |  | 70 | 75 | 0.80 |
| LV2791 | 71 | 65 | 0.90 |  | 70 | 88 | 0.87 |
| LV2805 | 68 | 64 | 0.94 |  | 46 | 75 | 1.00 |
| LV2953 | 66 | 66 | 0.95 |  | 53 | 80 | 0.94 |
| LV3094 | 67 | 68 | 0.96 |  | 53 | 87 | 0.96 |
| LV212 | 61 | 63 | 0.98 |  | 42 | 73 | 1.00 |

No.: Number
