# Supplementary material for: Genomic Comparison Among Global Isolates of L. interrogans Serovars Copenhageni and Icterohaemorrhagiae Identified Natural Genetic Variation Caused by an Indel
Source: Front Cell Infect Microbiol. 2018 Jun 19;8:193. doi: 10.3389/fcimb.2018.00193 (PMC6018220; doi:10.3389/fcimb.2018.00193)
Supplement: Table S7 — Primers employed for qRT-PCR. [file Table_7.DOCX]

**Table S7.** Primers employed for qRT-PCR

| **Primer** | **Sequence 5’- 3’** |
| --- | --- |
| 12008T1F | TCAAAGTGAAAATCGAGTTGCTT |
| 12008T1R | GGGGGATCATTCTCAGGA |
| 12008T2F | CATATGATTCCGGGCTTGAG |
| 12008T2R | CCGTCATCTATTTCGGCTTT |
| Sang. Seq F | TAGGTTGGCACGAAGGTTCT |
| Sang. Seq R | TTTTTCCGGGAACTCCAAC |
