# Supplementary material for: Genomic Comparison Among Global Isolates of L. interrogans Serovars Copenhageni and Icterohaemorrhagiae Identified Natural Genetic Variation Caused by an Indel
Source: Front Cell Infect Microbiol. 2018 Jun 19;8:193. doi: 10.3389/fcimb.2018.00193 (PMC6018220; doi:10.3389/fcimb.2018.00193)
Supplement: Table S8 — In vivo testing of virulence for L. interrogans serovar Icterohaemorrhagiae strains. [file Table_8.DOCX]

**Table S8.** *In vivo* testing of virulence for *L. interrogans* serovar Icterohaemorrhagiae strains

| **Strain** | **N° of animals** | **Infection route** | **Dose^a^** | **Lethality** | **Days for death** |
| --- | --- | --- | --- | --- | --- |
| 201000456 | 3 | Intraperitoneal | 100 | 3/3 | 11, 11, 12 |
|  |  | Conjunctival | 10^8^ | 2/3 | 11, 13 |
| 201000458 |  | Intraperitoneal | 100 | 3/3 | 11, 12, 12 |
|  |  | Conjunctival | 10^8^ | 3/3 | 11, 11, 11 |

^a^ Number of leptospires
